# Liquid Biopsy and Nonendoscopic Biomarkers for Precision Early Detection of Esophageal Cancer: Evidence, Risk Stratification and Translational Challenges

**DOI:** 10.7150/jca.140909

**Published:** 2026-09-03

**Authors:** Fangzheng Han, Huaimin Liu

**Affiliations:** 1 The Second Clinical Medical College of Henan University of Chinese Medicine, Zhengzhou, Henan, China.; 2 Henan Cancer Hospital, Zhengzhou, Henan, China.

**Keywords:** esophageal cancer, liquid biopsy, cell-free DNA methylation, nonendoscopic biomarkers, early detection, risk stratification

## Abstract

Esophageal cancer remains one of the most lethal malignancies worldwide, largely because most tumors are detected after symptoms develop. Esophageal squamous cell carcinoma (ESCC) and esophageal adenocarcinoma (EAC) differ in epidemiology, precursor lesions and molecular biology, and therefore require subtype-specific early-detection pathways. This narrative critical review used a structured PubMed/MEDLINE search and classifies evidence by intended use: population risk triage for ESCC in high-incidence regions; targeted case finding for previously undiagnosed Barrett esophagus; surveillance and progression prediction in established Barrett esophagus; diagnostic evaluation of symptomatic or referred patients; and multicancer early-detection studies as indirect contextual evidence. We evaluate nonendoscopic cell-collection devices, cfDNA methylation and fragmentomics, mutation-based circulating tumor DNA assays, circulating RNA and extracellular vesicles, autoantibodies, metabolomic and microbiome signals, and clinical risk models. Although many clinically diagnosed or case-control series report high AUROC values, such estimates often overstate performance in asymptomatic populations, where low prevalence sharply limits positive predictive value. The most credible near-term role is calibrated, risk-stratified triage that concentrates endoscopy on individuals most likely to harbor early cancer or high-grade precursors. Increased detection yield, favorable modeling and implementation feasibility are not equivalent to clinical utility; utility requires evidence that biomarker-guided care improves meaningful outcomes at acceptable cost and harm. Integrated multiomic testing remains a proposed future strategy rather than a prospectively validated esophageal-cancer screening platform.

## 1. Introduction

Esophageal cancer is a leading cause of cancer death. GLOBOCAN 2020 estimated 604,100 new cases and 544,100 deaths worldwide and projected approximately 957,000 new cases and 880,000 deaths by 2040 if incidence rates remain stable 1. The Global Burden of Disease (GBD) 2021 analysis estimated more than 570,000 incident cases and 356,000 deaths 2. These estimates should not be treated as directly comparable: GLOBOCAN and GBD use different calendar years, data inputs, cause-of-death redistribution procedures and statistical modeling frameworks. Survival is dominated by stage at diagnosis. Because early esophageal neoplasia is typically asymptomatic, many patients present with locally advanced or metastatic disease, whereas localized cancers and high-grade precursor lesions treated before invasion have a far more favorable prognosis. Detecting disease early, or intercepting precursors, is therefore central to esophageal-cancer control in appropriately defined high-risk populations 1,2.

The diagnostic reference standard, upper gastrointestinal endoscopy, is accurate; a meta-analysis of screening tests for squamous disease estimated pooled sensitivity and specificity for peroral endoscopy of 0.94 and 0.92, respectively 3. However, endoscopy is invasive, resource intensive, operator dependent and unattractive for population-wide screening, and adherence is limited even among identified high-risk groups. This mismatch between an accurate diagnostic test and the practical requirements of population screening is the fundamental problem that motivates the search for minimally invasive alternatives deliverable at scale.

**Figure 1:**
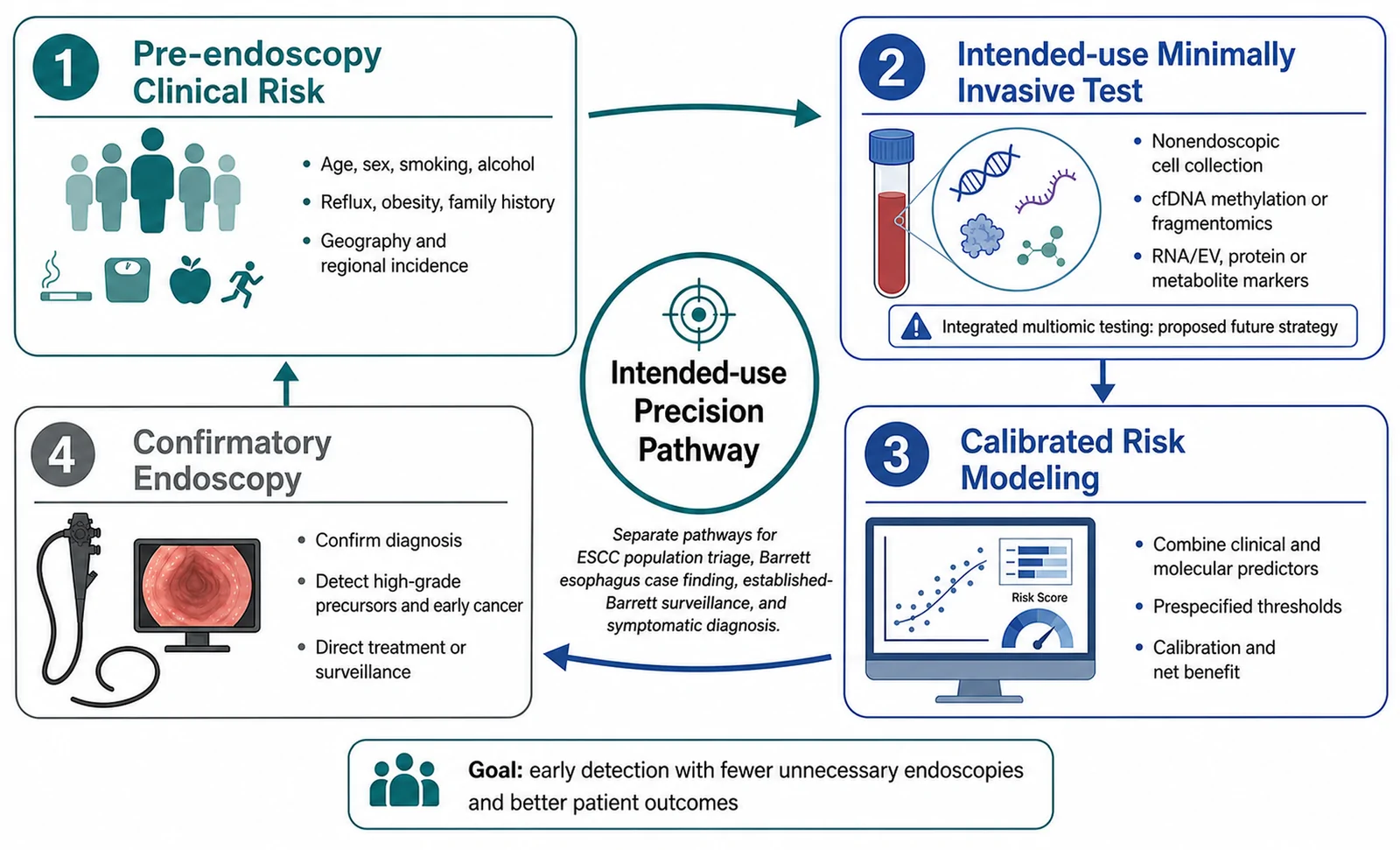
Sequential, intended-use framework for precision early detection. Pre-endoscopy clinical risk informs selection for an intended-use minimally invasive test; clinical and molecular data then generate a calibrated absolute-risk estimate linked to a prespecified endoscopic-referral threshold. Current evidence supports specific assays and devices, whereas integrated multiomic testing remains a proposed future strategy. ESCC population triage, targeted Barrett-esophagus case finding, established-Barrett surveillance and symptomatic diagnosis are separate pathways.

Liquid biopsy, understood broadly as the interrogation of tumor-derived or host-response analytes in blood, saliva or other accessible fluids, together with nonendoscopic cell-collection devices, offers a route to such alternatives. It is more useful, however, to conceive of these platforms not as a single blood test for cancer but as inputs to a risk-stratified triage strategy that concentrates endoscopy on those most likely to benefit (Figure 1). Under this framing the relevant question is not only whether a biomarker can separate cancer from health in a case series, but whether a biomarker-informed decision rule, applied prospectively to an asymptomatic population, detects more early cancers and high-grade precursors while sparing low-risk individuals' unnecessary endoscopy. This review critically appraises the current evidence for minimally invasive early detection of esophageal cancer, emphasizing throughout the distinctions between the squamous and adenocarcinoma pathways, between biomarker discovery and validation, and between diagnostic accuracy and demonstrated clinical utility. Where the term multiomic is used, we apply it cautiously, because the literature to date largely comprises separate analyte streams rather than prospectively validated integrated tests.

## 2. Review methods and evidence classification

This narrative critical review used a structured search of PubMed/MEDLINE via the NCBI Entrez interface. The principal search was conducted on July 8, 2026 and updated on August 18, 2026. Searches combined terms for esophageal cancer, ESCC, EAC and Barrett esophagus with concepts covering early detection, screening, surveillance, Cytosponge and other nonendoscopic sampling, liquid biopsy, cfDNA, ctDNA, DNA methylation, fragmentomics, circulating RNA, extracellular vesicles, autoantibodies, proteomics, metabolomics, microbiome, risk prediction, machine learning, calibration, clinical utility and cost-effectiveness. English-language articles published from 2015 through August 2026 were prioritized; older landmark studies, validation frameworks and reporting guidelines were retained when methodologically important.

Eligible evidence included randomized or pragmatic trials, prospective cohorts, nested case-control studies with prediagnostic specimens, diagnostic-accuracy studies, externally validated prediction models, systematic reviews, major guidelines and large translational biomarker studies relevant to early detection. Purely therapeutic studies, unvalidated small discovery reports without clear early-detection relevance, non-English reports and claims that could not be verified against a primary source were excluded. Titles and abstracts were screened for relevance and retained quantitative results were checked against the cited report. When multiple reports evaluated the same platform, representative studies were selected according to intended use, subtype specificity, early-stage relevance, study-design strength, independence of validation and maturity of implementation evidence. This approach was not designed as a systematic review, and no claim of exhaustive evidence coverage or PRISMA-style study accounting is made.

Evidence was classified by tumor subtype (ESCC, EAC/Barrett pathway, mixed or not reported), biomarker class, study design, specimen timing and validation status. Intended use was categorized as: (1) population risk triage for ESCC in high-incidence regions; (2) targeted case finding for previously undiagnosed Barrett esophagus; (3) surveillance or progression prediction in established Barrett esophagus; (4) diagnostic evaluation of symptomatic or clinically referred patients; or (5) multicancer early-detection evidence used only as indirect context. Diagnostic studies were appraised qualitatively using QUADAS-2 domains and prediction models using PROBAST domains, focusing on participant selection, index-test conduct, reference standard, timing, analysis, calibration and applicability 69,87. We report domain-based concerns rather than a numerical quality score. STARD, TRIPOD+AI and REMARK are treated as reporting guidance for diagnostic-accuracy, prediction-model and prognostic-marker studies, respectively 73,85,88.

## 3. Global burden and heterogeneity of esophageal cancer

A central obstacle to a universal early detection biomarker is that esophageal cancer is not one disease. Squamous cell carcinoma (ESCC) accounted for approximately 85% of cases globally in 2020, and adenocarcinoma (EAC) for approximately 14% 1. Their epidemiology diverges sharply. Registry-based apportionment of the 2018 global burden estimated the highest ESCC incidence in Eastern Asia (age-standardized rate 11.1 per 100,000), whereas adenocarcinoma rates were highest in Northern Europe (age-standardized rate 3.5 per 100,000) 4. Longitudinal international analyses show squamous incidence falling in about half of male populations while adenocarcinoma incidence rises in nearly a third of countries, a pattern attributed to a mix of birth-cohort and period effects 5. ESCC in endemic regions is linked to tobacco and alcohol, hot-beverage consumption, poor nutritional status and other environmental exposures, whereas EAC is driven by gastroesophageal reflux disease, obesity and its precursor lesion, Barrett esophagus 6,7.

These clinical distinctions are mirrored at the molecular level. Integrated genomic characterization of 164 esophageal carcinomas showed that ESCC and EAC are molecularly distinct: squamous tumors harbor frequent amplification of CCND1 and SOX2 and/or TP63, whereas adenocarcinomas are enriched for ERBB2, VEGFA and GATA4/GATA6 amplification and closely resemble the chromosomally unstable subtype of gastric adenocarcinoma 8. The practical implication is that biomarkers, sampling strategies and screening programs optimized for one subtype cannot be assumed to transfer to the other. Two corollaries follow. First, the global burden, still dominated by ESCC and concentrated in the Asian and East African esophageal-cancer belts, should not be conflated with the numerically smaller but rising adenocarcinoma epidemic of high-income countries 1,4. Second, the divergent tissue biology of the two subtypes shapes what is shed into blood and other fluids, so that the analyte classes, and the reference ranges, that perform well for squamous disease need independent development for adenocarcinoma (Figure 3).

## 4. Current approaches to early detection and surveillance

For ESCC, organized screening is largely confined to high-incidence regions, most extensively in China, and relies on endoscopy, frequently augmented by Lugol chromoendoscopy to highlight unstained dysplastic mucosa. Community-based and cohort evidence supports a mortality benefit. A multicenter cohort of 637,500 people reported that, compared with controls, upper gastrointestinal cancer incidence and mortality fell by 23% (relative risk 0.77, 95% CI 0.74-0.81) and 57% (relative risk 0.43, 95% CI 0.40-0.47) among those actually screened, and by 14% and 31% among all those invited 9. A cluster-randomized trial of one-time endoscopic screening detected predominantly early-stage lesions 10, and a large community-based cluster-randomized trial reported a 22% reduction in upper gastrointestinal cancer death in high-risk areas over 7.5 years (risk ratio 0.78, 95% CI 0.66-0.91) 11. The prognostic weight of precursor histology is well established: in a prospective cohort, cumulative ESCC incidence over a median 8.5 years was 15.5% for severe dysplasia or carcinoma in situ, 4.5% for moderate and 1.4% for mild dysplasia 12, and long-term multicenter data confirm that baseline dysplasia grade stratifies subsequent cancer risk over more than a decade of follow-up 13. Nonetheless, endoscopic screening is resource intensive and its incremental yield falls at younger ages, so that programs must weigh the number needed to screen and the age at which to begin; these considerations, rather than test accuracy alone, frequently determine cost-effectiveness.

For EAC, early detection centers on identifying and surveilling Barrett esophagus, the only recognized precursor. The absolute progression risk from non-dysplastic Barrett esophagus is low; a nationwide cohort estimated the annual risk of adenocarcinoma at 0.12% (95% CI 0.09-0.15), with an 11.3-fold relative risk versus the general population and a substantially higher risk in the presence of low-grade dysplasia 14. A meta-analysis reported an EAC incidence of 5.5 per 1,000 person-years and estimated that surveillance-detected cancers carry lower relative mortality than those detected outside surveillance 15, and abnormal p53 immunohistochemistry predicts progression, with a hazard ratio of approximately 5 in a validation cohort, offering an objective adjunct to subjective dysplasia grading 16. Contemporary guidelines from the American College of Gastroenterology, the American Gastroenterological Association and the European Society of Gastrointestinal Endoscopy accordingly recommend risk-tailored screening and surveillance, increasingly acknowledging nonendoscopic tools 17-19. Yet symptom-based entry criteria are themselves a weak filter: in an unreferred primary-care population, guideline criteria requiring reflux symptoms had sensitivities of only 39-43% for prevalent Barrett esophagus, because many affected individuals do not report typical symptoms 20. Surveillance is further limited by sampling error, interobserver variability in dysplasia grading, and the risk of overdiagnosis and overtreatment of indolent disease, given the low absolute progression rate. The low progression rate also magnifies the influence of lead-time and length biases when screening is evaluated by survival rather than by mortality or stage-specific incidence, so that apparent gains can be illusory. These limitations, common in principle to both subtypes, define the niche for minimally invasive tests: their purpose is not to replace endoscopy but to decide, more efficiently and acceptably than symptom-based criteria, who should undergo it.

## 5. Nonendoscopic sampling and liquid biopsy as early detection platforms

Nonendoscopic cell-collection devices were the first minimally invasive tools to reach large clinical trials, and they illustrate both the promise and the boundaries of the approach. The Cytosponge, an ingestible sponge-on-a-string that samples the esophageal mucosa for the Barrett marker trefoil factor 3, achieved an overall sensitivity of 79.9% for Barrett esophagus in a multicenter diagnostic study, rising to 87.2% for segments of 3 cm or longer, with a specificity of 92.4% 21. In the pragmatic, cluster-randomized BEST3 trial of 13,514 patients, offering Cytosponge-TFF3 in primary care increased Barrett detection roughly tenfold relative to usual care and identified dysplastic Barrett esophagus and early cancers that would otherwise have been missed 22. A meta-analysis of six studies (3,438 participants) estimated pooled sensitivity of 0.81 and specificity of 0.89 for any-length Barrett esophagus 23. Two points deserve emphasis. First, the Cytosponge addresses the Barrett-to-adenocarcinoma pathway and is not a validated tool for squamous disease; distinct swallowable-sponge cytology combined with machine learning is being developed for ESCC screening, with one nationwide multicohort study reporting an area under the curve of 0.960 for an automated cytology model 24. Second, the value of the device lies increasingly in molecular and computational refinement rather than in cytology alone.

Molecular refinement of nonendoscopic samples has improved diagnostic yield and enabled triage. Methylated-DNA marker panels applied to sponge or balloon samples have achieved high diagnostic accuracy for Barrett esophagus 25,26, while a Cytosponge biomarker panel has prioritized patients with established Barrett esophagus for endoscopy and deep-learning triage has reduced pathology workload 27,28. These uses must remain distinct. BEST3 evaluated targeted case finding for previously undiagnosed Barrett esophagus in selected primary-care patients, whereas biomarker panels in known Barrett esophagus address surveillance or progression risk. Increased detection, high negative predictive value and operational feasibility are valuable implementation findings, but they do not by themselves demonstrate reduced EAC mortality or clinical utility. Nonendoscopic testing is best understood as an intended-use-specific triage layer followed by confirmatory endoscopy; acceptability, throughput and primary-care feasibility are therefore relevant alongside accuracy.

Blood-based liquid biopsy extends this logic to a peripheral sample. Multicancer early-detection (MCED) studies provide methodological context but are not esophageal-cancer validation. Targeted methylation classifiers in the Circulating Cell-free Genome Atlas achieved specificity around 99.3%-99.5%, with sensitivity strongly dependent on stage 29,30. These studies illustrate two binding constraints: early-stage sensitivity and the low prevalence of disease in the intended screening population. The major platforms are summarized by subtype and intended use in Table 1; Table 2 reports design, sample composition, threshold handling, early-stage performance and validation for representative studies; and a detailed evidence matrix is provided in Table S1.

Screening performance depends on pre-test probability. Positive predictive value (PPV) is calculated as sensitivity × prevalence / [sensitivity × prevalence + (1 - specificity) × (1 - prevalence)]. At a prevalence of 0.1%, a test with 90% sensitivity and 95% specificity has a PPV of approximately 1.8%; at 0.5% prevalence, PPV is approximately 8.3%. Thus, more than 90% of positive results may be false positives even when sensitivity and specificity appear favorable. This mathematical constraint supports sequential risk enrichment and makes the number of downstream endoscopies, complications, anxiety and cost essential evaluation outcomes.

## 6. Circulating DNA methylation, mutation-based ctDNA and fragmentomic biomarkers

Among blood-based analytes, total cell-free DNA (cfDNA) must be distinguished from the tumor-derived fraction, circulating tumor DNA (ctDNA). Mutation-based ctDNA detection is constrained in early disease by very low tumor fractions and by clonal hematopoiesis of indeterminate potential. In matched plasma and white-blood-cell sequencing, 81.6% of circulating variants in controls and 53.2% in patients with cancer had features of clonal hematopoiesis rather than tumor origin 31. Consequently, matched leukocyte or buffy-coat sequencing is a mandatory technical control for mutation-based early-detection assays; plasma-only mutation calls risk clinically important false positives. cfDNA methylation and fragmentomic signals measure different biological features and should not be conflated with mutation detection. Methylation offers a broader early signal and is comparatively less affected by hematopoietic point mutations. In ESCC, a multimodal cfDNA-methylome model detected 87% of cancers and 62% of precancerous lesions at greater than 95% specificity in validation cohorts 32. A multicenter prospective three-marker model achieved 85.5% sensitivity and 95.3% specificity overall, but sensitivity was 56% for stage 0 and 77% for stage I 33. A double-blinded prospective OTOP2/KCNA3 assay reported stage I sensitivity of 78.5% (95% CI 69.1%-85.6%) 34. In EAC, evidence is less mature and often derives from tissue or endoscopic brushings rather than screening blood samples 37.

Across these studies, overall sensitivity is consistently higher than sensitivity for stage 0-I disease. The fraction of tumor-derived molecules scales with tumor burden, and clinically diagnosed or case-control cohorts enrich the very cases that are easiest to detect. Their headline AUROC values should therefore be interpreted as diagnostic-development estimates, not expected screening performance. Fragmentomics analyzes cfDNA fragment sizes, end motifs and genome-wide patterns that reflect nucleosomal architecture and tissue of origin; it is an orthogonal signal rather than a synonym for ctDNA mutation testing. Genome-wide fragmentation distinguished several cancers from healthy controls with an AUROC of 0.94 at 98% specificity, although esophageal cancer was not a primary focus 38, and an ESCC-specific stacked model has undergone independent-cohort evaluation 39. PanSeer provides rare prediagnostic evidence, but it was a retrospective nested case-control analysis across five cancers and did not report esophageal-specific performance; it should be treated as indirect proof of principle 40.

## 7. RNA, extracellular vesicle and autoantibody biomarkers

Circulating RNA species and their vesicular carriers constitute a second major analyte class. MicroRNAs are attractive because of their stability in blood and other fluids. Early serum microRNA fingerprints distinguished ESCC from controls 41, and a diagnostic meta-analysis of 85 studies estimated pooled sensitivity and specificity of circulating microRNA for esophageal cancer of 0.82 and 0.84 with an area under the curve of 0.89 42. However, the meta-analysis of ESCC screening tests found microRNA to have more modest pooled sensitivity and specificity (0.77 and 0.78) than endoscopy, underlining that single-analyte RNA tests are unlikely to suffice alone 3. Long non-coding RNAs such as plasma POU3F3, alone or combined with a protein marker, have shown high diagnostic performance including for early-stage ESCC 43. For EAC, a nested case-control study using a whole-transcriptome assay found many differentially expressed circulating microRNAs but only moderate discrimination (area under the curve 0.62), a useful reminder that adenocarcinoma biomarkers have generally lagged behind squamous ones and that convenience case-control designs do not guarantee strong performance 44.

Extracellular vesicles (EVs) protect molecular cargo from degradation and may enrich tumor-associated signal. A six-miRNA salivary EV signature identified stage I/II ESCC with approximately 90% sensitivity and 89%-91% specificity across training and validation cohorts 45, and a glycosylated EV-miRNA score reported AUROCs of 0.957-0.980 46. However, these cohorts were enriched diagnostic samples rather than population-screening cohorts, so the estimates require prospective intended-use validation. Pre-analytical variation is a major barrier: serum and plasma yield different EV-miRNA profiles 47; hemolysis releases erythrocyte-enriched miR-16 and miR-451a and can create artifactual differences 89; and residual platelets or platelet-derived vesicles alter circulating-miRNA abundance. Translation therefore requires standardized collection tubes, prompt processing, documented centrifugation and platelet depletion, hemolysis assessment, storage controls and prespecified quality-control thresholds.

Tumor-associated autoantibodies represent a third class, exploiting the amplified and stable humoral response to early neoplastic antigens. Panels of autoantibodies have been developed and validated for ESCC: a six-antigen panel reported sensitivity of 51-57% at specificity of 95-96%, including detection of early-stage disease 48. The defining feature of autoantibody testing, however, is high specificity with limited sensitivity: a systematic review of 45 studies found that single autoantibodies had a median specificity of 98.3% but median sensitivity of only 26.7% 49, and the ESCC screening-test meta-analysis estimated pooled autoantibody sensitivity of just 0.45 at specificity 0.91 3. Autoantibody panels are therefore best regarded as high-specificity rule-in components of a composite strategy rather than stand-alone screening tests 50.

## 8. Proteomic, metabolomic, lipidomic and microbiome signals

Beyond nucleic acids, proteomic, metabolomic and lipidomic signatures capture tumor metabolism and host response. Clinically diagnosed ESCC series have reported high AUROCs, including 0.984 for a serum metabolite panel and 0.930 for a urine panel 51, while serum lipidomic panels have reported AUROCs of 0.82-0.97 52. These estimates come from enriched diagnostic cohorts and should not be interpreted as population-screening performance. Prediagnostic performance is more modest and time-dependent: a nested case-control metabolic risk score had an AUROC of 0.815 overall and 0.868 within one year of diagnosis 53, while a UK Biobank metabolite model had an AUROC around 0.70 for upper gastrointestinal cancer 54. Reviews identify moderate-to-poor reporting quality and few genuinely independent validations 55. For EAC and Barrett esophagus, inflammatory and metabolic biomarkers linked to obesity show modest associations and are more plausible as risk-stratification inputs than stand-alone diagnostic tests 56.

The oral and esophageal microbiome has emerged as a biologically plausible and, importantly, sometimes prediagnostic signal. In prospective nested case-control studies, prediagnostic oral microbiome composition was associated with subsequent risk of both EAC and ESCC, with specific periodontal pathogens associated with higher risk 57, and a multi-species oral microbial model discriminated severe squamous dysplasia and above from controls with an area under the curve of 0.89 58. Microbiome alterations also accompany progression along the Barrett-to-adenocarcinoma sequence, with reduced diversity and shifts from Firmicutes toward Proteobacteria in high-grade dysplasia and cancer 59. Because such signatures are measured in prediagnostic samples in some studies, they partially escape the case-control spectrum bias that limits many other biomarker classes; however, sample sizes remain small, external validation is limited, and causal versus consequential relationships are unresolved. Integrated analyses combining, for example, salivary exosomal proteomic and lipidomic features illustrate the direction of travel toward multi-analyte panels, but reported near-perfect discrimination in small single-center cohorts should be treated as hypothesis-generating pending independent validation.

## 9. Risk prediction and precision screening

### 9.1 Pre-endoscopy population risk models

Pre-endoscopy models use variables available before any invasive procedure and can therefore select individuals for initial testing. ESCC models based on demographic, lifestyle and symptom variables have reported development AUROCs around 0.81 60, but external validation of published models produced C statistics from 0.51 to 0.74 62. A cohort-derived model using age, sex, smoking, alcohol and body-mass index achieved an AUROC of approximately 0.70 with calibration and decision-curve analysis reported in external data 63. For the EAC pathway, population models based on age, sex, reflux, obesity and smoking can identify higher-risk strata 64, while polygenic and electronic-health-record models may add information 66,67. Their intended use is targeted case finding or initial referral, not surveillance after endoscopy.

### 9.2 Post-endoscopy lesion models and Barrett progression models

Models that require endoscopic findings operate later in the pathway. The Han et al. ESCC models included lesion counts, lesion size and dysplasia identified at baseline endoscopy and predicted incident ESCC during follow-up; they therefore support post-endoscopy follow-up decisions rather than initial population triage 61. Among 104,129 participants, 252 and 61 incident ESCC cases occurred in the derivation and validation cohorts, and validation C statistics were 0.90-0.91. Models predicting progression in established Barrett esophagus likewise inform surveillance intervals after the precursor has been diagnosed and should not be combined with case-finding models for previously undiagnosed Barrett esophagus 65.

### 9.3 Biomarker-informed second-stage referral

A biomarker can enter a clinical pathway in two credible ways. In a sequential design, an inexpensive pre-endoscopy clinical score first enriches risk, and a minimally invasive biomarker is then applied to the enriched group before endoscopic referral. In a joint model, clinical and molecular predictors are combined into one absolute-risk estimate. Either approach requires recalibration in the intended population, a prespecified decision threshold linked to endoscopic capacity and harms, and comparison with a clinical-only strategy. A biomarker should be retained only if it improves calibration or net benefit, not merely AUROC.

### 9.4 Calibration, overconfidence and clinical decision value

Discrimination is necessary but insufficient. Flexible machine-learning algorithms can produce extreme, overconfident probabilities in sparsely represented tails even when ranking performance appears strong. External calibration plots, calibration-in-the-large, calibration slopes and decision-curve analysis across clinically relevant thresholds are therefore essential before deployment 70. A formal appraisal using PROBAST found all reviewed esophageal risk models at high risk of bias, largely because of analysis-stage limitations, limited calibration and insufficient external validation 68,69. Precision screening should be judged by early cancers detected, unnecessary endoscopies avoided and net benefit in the intended population, not by development-cohort discrimination alone.

## 10. Translational pathway from biomarker discovery to clinical implementation

**Figure 2:**
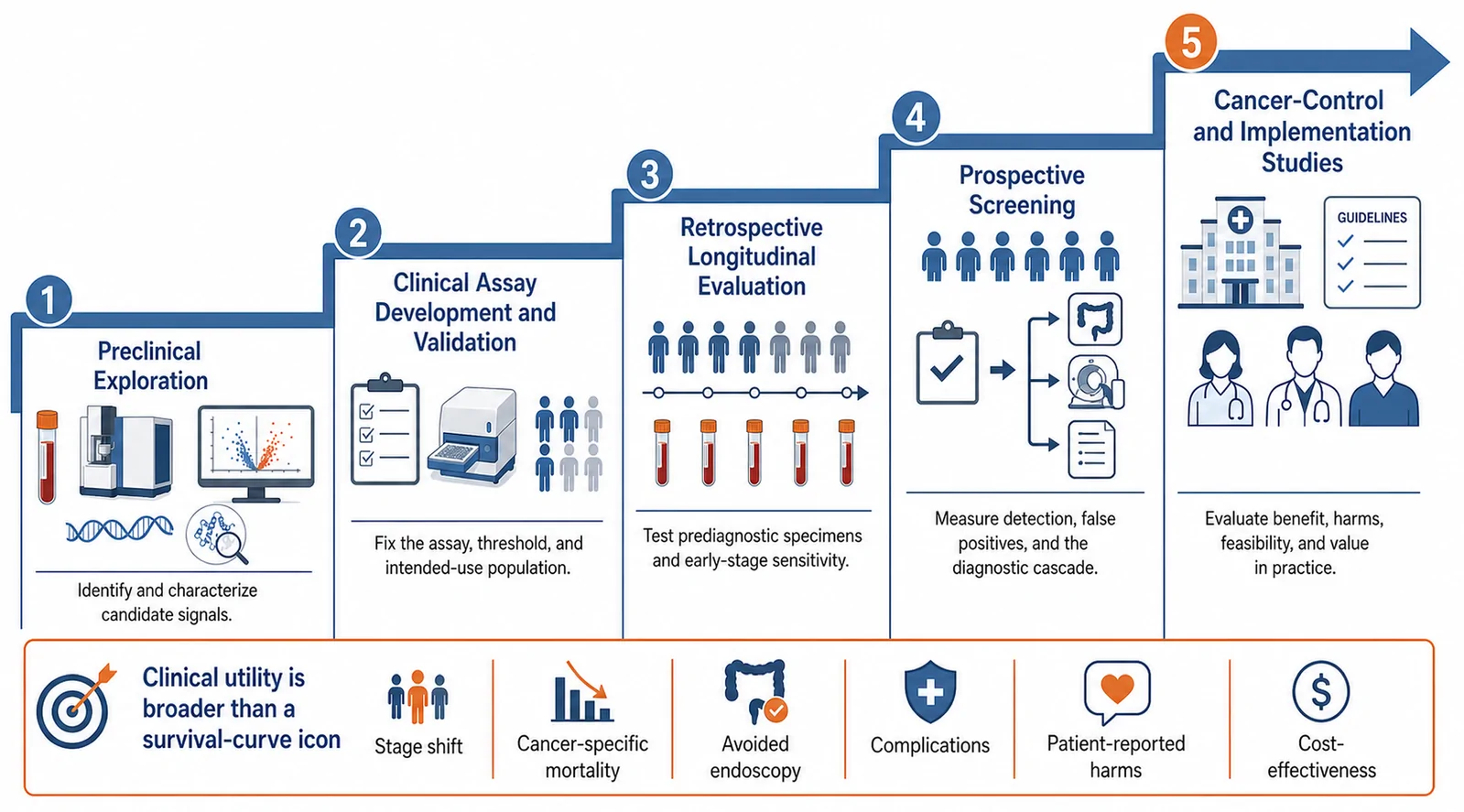
Five-phase evidence pathway for early-detection biomarkers, adapted from the classic framework of Pepe et al. 71: preclinical exploration; clinical assay and validation; retrospective longitudinal evaluation with prediagnostic specimens; prospective screening; and cancer-control/implementation studies. Clinical utility encompasses stage shift, cancer-specific mortality, avoided endoscopy, complications, patient-reported harms and cost-effectiveness.

The path from a promising biomarker to practice is sequential (Figure 2). The classic early-detection framework comprises preclinical exploration, clinical assay development and validation, retrospective longitudinal evaluation with prediagnostic specimens, prospective screening, and cancer-control studies that test whether screening reduces disease burden 71. Prospectively collected specimen reference sets help avoid convenience-sampling bias 72. Reporting should follow STARD for diagnostic-accuracy studies, TRIPOD+AI for regression- or machine-learning prediction models, and REMARK for prognostic tumor-marker studies; QUADAS-2 and PROBAST are risk-of-bias tools rather than reporting checklists 69,73,85,87,88.

Three levels of evidence remain conceptually distinct. Analytical validity concerns measurement reliability and reproducibility; clinical validity concerns performance in the intended-use population; and clinical utility concerns whether acting on the result improves meaningful outcomes at acceptable cost and harm. Diagnostic yield, guideline acknowledgement, regulatory designation, a high NPV and modeled cost-effectiveness are not interchangeable with utility. Cytosponge-TFF3 has randomized evidence for increased Barrett-esophagus case finding and favorable economic modeling 22,77,78, but reduced EAC mortality has not been demonstrated. Likewise, PATHFINDER shows that false-positive signals can lead to prolonged diagnostic resolution and additional procedures 79. Relevant utility endpoints include stage shift, cancer-specific mortality, avoided endoscopy, complications, patient-reported harms and cost-effectiveness, assessed in pragmatic intended-use trials.

## 11. Distinct challenges for ESCC and EAC

**Figure 3:**
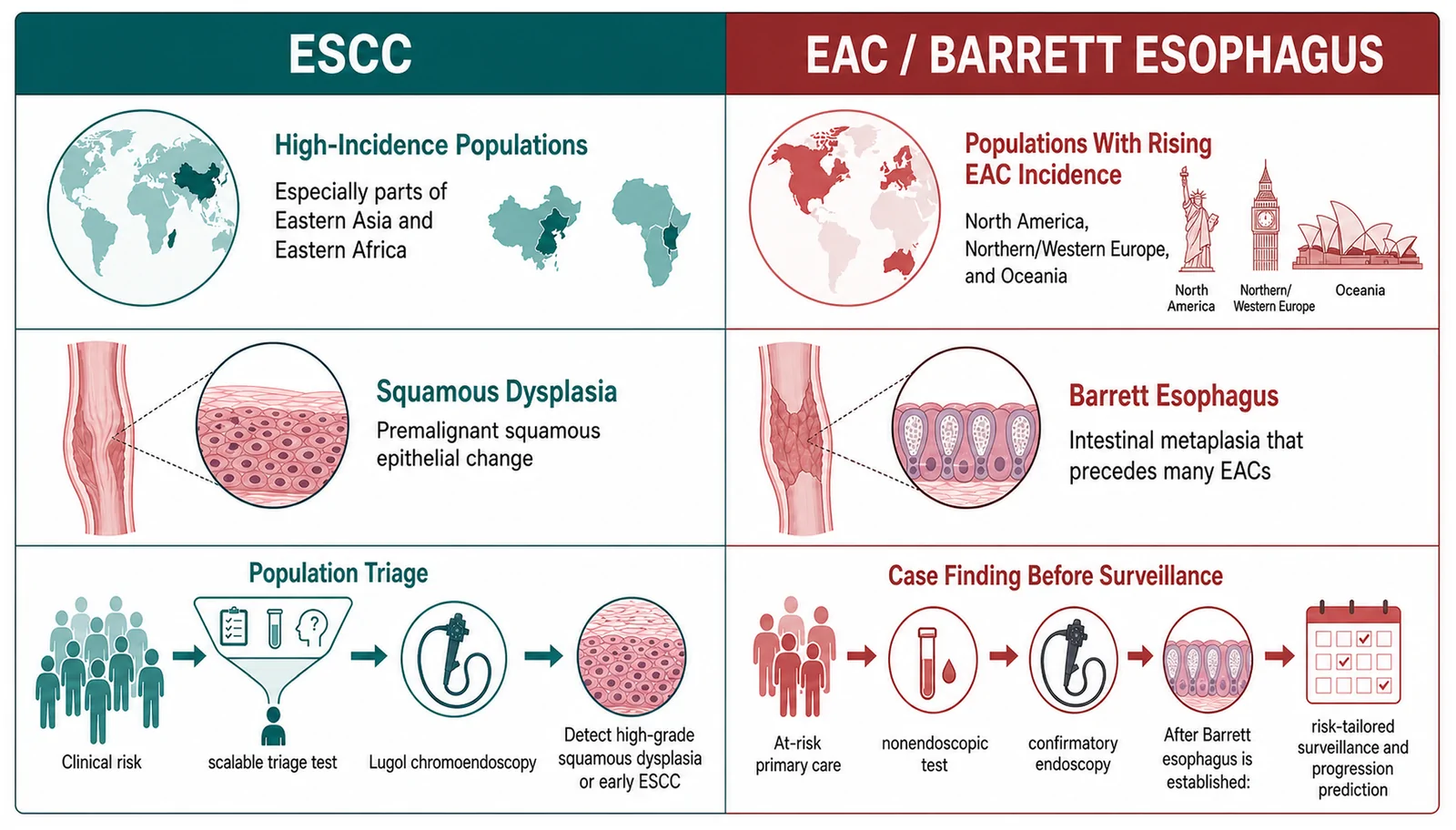
Subtype-specific early-detection pathways. ESCC programs generally target population triage in high-incidence regions. The EAC pathway begins with targeted case finding for previously undiagnosed Barrett esophagus in at-risk adults, followed by confirmatory endoscopy and, only after Barrett esophagus is established, risk-tailored surveillance and progression prediction. The two pathways require separate populations, tests, thresholds and endpoints.

ESCC and EAC require different early-detection programs (Figure 3). ESCC programs generally target asymptomatic populations in high-incidence regions and prioritize low cost, scalability and detection of squamous dysplasia; endoscopic screening has outcome evidence in these settings 9-11. EAC programs must first identify previously undiagnosed Barrett esophagus among at-risk adults, often through targeted case finding, before surveillance and progression prediction can occur in patients with established Barrett esophagus. These are separate intended uses with different prevalence, thresholds and endpoints. EAC incidence is elevated in North America, Northern and Western Europe and Oceania, but the label 'Western countries' is overly categorical. Subtype-specific biomarker discovery, validation, implementation and health-economic evaluation are required, and mixed-population results should report ESCC and EAC separately whenever possible.

## 12. Methodological pitfalls and reasons biomarkers fail

The gap between discovery-cohort accuracy and real-world performance has identifiable causes. Spectrum bias is central: clinically diagnosed, often advanced cases and healthy controls do not reproduce the asymptomatic population, early-lesion spectrum or comorbidity distribution encountered in screening. Accordingly, the high AUROCs in Sections 6-8 should be read as development estimates unless a study used an intended-use prospective or prediagnostic design. Control selection, specimen timing, stage distribution, threshold selection and independent validation are now displayed in Table 2. Prediagnostic specimen sets and prespecified analyses are needed to reduce these biases 72,81.

Overfitting arises when high-dimensional features greatly outnumber outcome events, producing unstable models whose apparent performance shrinks in new data 82,83. Biological and pre-analytical noise compounds the problem: mutation-based assays require matched leukocyte sequencing to remove clonal-hematopoiesis variants 31, while miRNA/EV studies require rigorous hemolysis and platelet controls 47,89. Finally, diagnostic accuracy is often conflated with utility. Low prevalence can make PPV poor even at apparently favorable sensitivity and specificity, and downstream diagnostic cascades can cause harm or consume endoscopic capacity. Robust evaluation therefore requires prespecified thresholds, intended-use cohorts, independent validation, calibration and net-benefit assessment, followed by pragmatic studies of stage shift, mortality, avoided procedures, harms and cost 70.

## 13. Future directions

Three priorities follow. First, the strongest candidates should undergo adequately powered, subtype-specific validation in intended-use cohorts, with emphasis on stage 0-I disease, high-grade precursors and prediagnostic specimens. Mixed ESCC/EAC performance and clinically diagnosed case-control accuracy should no longer be treated as adequate screening evidence.

Second, biomarker studies should evaluate a complete decision pathway. Sequential and joint clinical-molecular models must report calibration, clinically prespecified thresholds and net benefit, and should follow the final TRIPOD+AI guideline 85. Integrated multiomic panels remain scientifically plausible, but must outperform simpler assays in independent data before added complexity is justified.

Third, strategies that survive validation require pragmatic evaluation of clinically meaningful outcomes, downstream procedures, patient-reported harms and cost-effectiveness. Discovery of new biomarker classes remains important, but immediate translational gains will depend on rigorous validation and implementation studies of the most credible existing candidates.

## 14. Conclusions

Minimally invasive early detection of esophageal cancer has a strong biological rationale, but evidence maturity varies by subtype and intended use. Nonendoscopic devices have randomized and implementation evidence for targeted Barrett-esophagus case finding, while much of the blood-biomarker literature is dominated by ESCC diagnostic cohorts. High AUROC values from case-control studies do not establish screening performance, and low prevalence can produce poor PPV and many unnecessary endoscopies. The most credible near-term role is a calibrated triage layer that integrates pre-endoscopy clinical risk with an intended-use biomarker and directs selected individuals to confirmatory endoscopy. ESCC population triage, Barrett-esophagus case finding, Barrett surveillance and symptomatic diagnosis must be evaluated separately. Important new biomarker classes may still emerge, but rigorous validation of the strongest current candidates, followed by pragmatic trials of meaningful outcomes, is the immediate translational priority.

## Supplementary Material

Supplementary table.

## Figures and Tables

**Table 1:** Major early-detection platforms and analytes, classified by subtype, intended use and translational readiness.

Platform / analyte	Source	Subtype / intended use	Representative evidence	Advantages	Limitations	Readiness
Endoscopy ± Lugol chromoendoscopy	Direct visualization / biopsy	ESCC population screening; EAC diagnosis and surveillance	3,9-11,17-19	Histologic confirmation; ESCC/upper-GI outcome evidence	Invasive, resource intensive; EAC-surveillance mortality benefit not established	Established reference standard; outcome evidence is setting-specific
Nonendoscopic device + molecular marker	Esophageal cells / DNA	Targeted BE case finding or established-BE triage	21,22,25-28	Primary-care delivery; randomized case-finding evidence	Not validated for ESCC; utility for cancer outcomes unproven	Advanced implementation evidence for defined Barrett-pathway uses
Swallowable sponge cytology + ML	Esophageal cytology	ESCC / EGJ high-grade-lesion triage	24	Prospective multicohort; high PPV relative to blood tests	Needs independent implementation and outcome validation	Emerging validation
cfDNA methylation	Plasma cfDNA	Mostly ESCC diagnostic evaluation; EAC evidence limited	32-37	Broad early signal; high specificity	Stage 0-I sensitivity lower; mixed subtype data often unreported	Emerging multicenter diagnostic validation
Mutation-based ctDNA	Plasma cfDNA + matched leukocytes	Diagnostic research; no validated population-screening use	31	Tumor-specific variants when true origin is confirmed	Low tumor fraction; CHIP false positives; buffy-coat sequencing mandatory	Early evidence
cfDNA fragmentomics	Plasma cfDNA	ESCC diagnostic research; MCED context	35,38,39	Genome-wide orthogonal signal; tissue-of-origin potential	Threshold and platform standardization; limited intended-use validation	Early to emerging validation
Circulating / salivary RNA	Serum, plasma, saliva	Predominantly ESCC diagnostic evaluation	41-45	Stable, accessible analytes	Hemolysis, platelet and normalization artifacts; EAC data limited	Early to emerging validation
Extracellular-vesicle cargo	Serum, plasma, saliva	Predominantly ESCC diagnostic evaluation	45-47	Protected cargo; multicohort signals	Isolation and pre-analytics not standardized	Emerging validation; utility unproven
Tumor-associated autoantibodies	Serum	ESCC rule-in adjunct	3,48-50	Stable, inexpensive, high specificity	Low sensitivity alone	Early adjunctive evidence
Proteomic / metabolomic / lipidomic	Serum, urine, saliva	Mostly ESCC diagnostic or prediagnostic risk research	51-56	Captures tumor and host response	High case-control AUROCs; few external validations	Early evidence
Microbiome	Saliva, mouthwash, tissue	ESCC/EAC risk research	57-59	Some prediagnostic data	Small samples; causality and transportability unresolved	Early evidence
Clinical / polygenic risk models	Clinical, EHR, genotype	Pre-endoscopy triage, post-endoscopy follow-up or BE progression (model-specific)	60-69	Scalable risk enrichment	Pathway position, calibration and external validity often unclear	Emerging but heterogeneous

Readiness definitions: Established = accepted reference standard with outcome evidence in at least one intended setting; Advanced implementation evidence = prospective/pragmatic or randomized evidence for a defined use, without proof of cancer-outcome benefit; Emerging validation = multicenter or prospective diagnostic validation without population-screening utility; Early evidence = discovery, internal validation or limited external validation. Categories refer to the stated intended use and are not interchangeable with regulatory status or guideline mention.

**Table 2:** Design, participant composition, threshold handling, performance, validation and applicability of selected early-detection studies.

Study	Subtype / intended use	Participants, cases and stage	Design, controls, timing and threshold	Performance (95% CI where reported)	Validation / risk of bias and applicability
Chen R 2020 9	ESCC / upper-GI population screening	N=637,500; 299,483 controls; 338,017 invited; 113,340 screened. Cancer/stage counts not reported in this summary.	Multicenter population cohort; endoscopy at baseline; outcomes by registry follow-up; no assay threshold.	Invited analysis: incidence -14%, mortality -31%; screened analysis: incidence -23%, mortality -57%.	Multicenter cohort; moderate confounding/self-selection risk; applicable to high-incidence Chinese settings.
He Z 2018 10	ESCC population screening	N=33,948; 17,151 screening and 16,797 control; 69.9% of detected high-grade lesions were early stage.	Cluster-randomized trial; Lugol endoscopy; preliminary stage outcome; no biomarker threshold.	Early-stage distribution 69.9%; mature mortality estimate not reported in this publication.	Randomized design; preliminary outcomes; setting-specific applicability.
Fitzgerald 2020 22	Targeted case finding for previously undiagnosed BE	13,514 randomized; analysis 6,388 usual care vs 6,834 intervention; BE 13 vs 140; four stage I cancers after positive testing.	Pragmatic cluster/individual RCT in selected primary-care patients; TFF3-positive test triggered endoscopy.	Adjusted BE detection rate ratio 10.6 (6.0-18.8).	Low-to-moderate bias; strong case-finding evidence, not universal screening or mortality utility.
Ross-Innes 2015 21	BE case finding / diagnostic accuracy	N=1,110; 647 known BE cases and 463 dyspepsia/reflux controls; cancer stage not applicable.	Multicenter case-control; Cytosponge-TFF3 before endoscopy; prespecified TFF3 interpretation.	Sensitivity 79.9% (76.4-83.0); specificity 92.4% (89.5-94.7); sensitivity 87.2% for BE ≥3 cm.	High spectrum-bias concern; clinically known BE cases overrepresent established disease.
Moinova 2018 25	BE case finding	Discovery/biological cohort n=173; independent validation n=149; balloon subset n=86 (50 BE, 36 controls).	Case-control biomarker development; endoscopy reference; cutoff established in training and applied to validation.	CCNA1+VIM sensitivity 90.3%; specificity 91.7% in balloon samples (CIs not reported in summary).	Independent validation but enriched case-control sampling; applicability concern for primary care.
Gao 2023 24	ESCC / EGJ high-grade-lesion population triage	N=17,498 across training, testing and community validation; subtype-specific case counts not reported in abstract.	Nationwide prospective multicohort; sponge cytology + epidemiology; model-defined risk threshold; endoscopy/histology reference.	Test AUROC 0.960 (0.937-0.977); sensitivity 94.5% (88.8-97.5); specificity 91.9% (91.2-92.5); PPV 18.4% (15.6-21.6).	Prospective multicohort with community validation; subtype-specific performance not reported.
Liu 2024 32	ESCC / precursor diagnostic development	460 cfDNA samples from nonmetastatic ESCC, precancerous lesions and matched controls; exact stage counts not reported in abstract.	WGBS case-control development with validation cohorts; threshold selected during model development.	AUROC increased from 0.90 to 0.99; sensitivity 87% for ESCC and 62% for precursors at >95% specificity.	Internal/held-out validation; high spectrum-bias and screening-applicability concerns.
Zhang R 2025 33	Mixed EC diagnostic evaluation	Model-verification cohort n=297; prospective multicenter cohort n=1,429; subtype/case counts not reported in abstract.	Three-MDM model developed before prospective evaluation; clinically recruited non-EC controls; specimen collected at diagnosis.	Sensitivity 85.5%; specificity 95.3%; stage 0 sensitivity 56%; stage I 77% (CIs not reported in abstract).	Prospective multicenter diagnostic validation; mixed-histology results not subtype-specific; screening utility untested.
Bian 2024 34	Mixed EC / HGIN diagnostic evaluation	N=1,116: 334 EC, 71 HGIN and 711 controls; subtype-specific counts not reported.	Double-blinded multicenter prospective study; OTOP2/KCNA3 assay; clinically recruited controls; locked test process reported.	Overall sensitivity 87.4% (83.4-90.6); specificity 93.3% (91.2-94.9); stage I sensitivity 78.5% (69.1-85.6).	Prospective multicenter validation; enriched prevalence and mixed-histology applicability concerns.
Chen X 2020 40	MCED contextual evidence (indirect)	605 asymptomatic samples: 191 later diagnosed with five cancers and 414 controls; plus 223 postdiagnosis cancers. Esophageal-specific n/stage not reported.	Retrospective nested case-control; prediagnostic plasma up to 4 years; cutoff locked before leave-out testing.	Prediagnostic sensitivity 95% (89-98); postdiagnosis sensitivity 88% (80-93); specificity 96% (93-98); not esophageal-specific.	Prediagnostic design but enriched sampling; no esophageal-specific validation or population PPV.
Li K 2023 45	ESCC diagnostic evaluation	Pilot n=54; discovery n=72; training n=342; internal validation n=207; external validation n=226; exact case/control counts by stage incompletely reported here.	Prospective multicohort salivary-EV miRNA model; diagnostic controls; threshold developed in training.	Early-stage sensitivity/specificity: 92.0%/89.2% training; 90.3%/91.0% internal; 91.1%/88.1% external (CIs not reported in summary).	Independent external cohort; case-control spectrum and pre-analytical applicability concerns.
Han J 2023 61	Post-endoscopy ESCC follow-up model	N=104,129; derivation 59,481 with 252 incident ESCC; validation 44,648 with 61 incident ESCC.	Prospective multicenter follow-up; predictors include baseline endoscopic lesion counts/dysplasia; thresholds reported in score table.	Validation Harrell C: model A 0.90 (0.87-0.93); model B 0.91 (0.88-0.95).	Temporal/geographic validation; not usable for initial pre-endoscopy triage; few events in validation.

Abbreviations: BE, Barrett esophagus; EC, esophageal cancer; EGJ, esophagogastric junction; HGIN, high-grade intraepithelial neoplasia; MDM, methylated-DNA marker; NR/not reported means that the requested subtype-, stage- or threshold-specific value was not available in the cited report or its abstract and was not inferred. 'External validation' is reserved for data genuinely independent of model development and threshold selection.

**Table 3:** Research priorities for translating liquid biopsy and nonendoscopic biomarkers into intended-use precision screening.

Domain	Current gap	Recommended action	Ideal study design	Key methodological requirement
Validation design	Reliance on case-control discovery that overstates screening performance	Prospectively validate the strongest existing candidates before further discovery	Nested case-control within screening or population cohorts using prediagnostic biobanked samples	Prespecified analysis; PRoBE-compliant, prediagnostic specimen reference sets
Subtype specificity	Pooling of ESCC and EAC obscures divergent biology	Develop and report subtype-specific panels and thresholds	Separate ESCC and EAC discovery and validation cohorts	Subtype-stratified performance reporting
Early-stage sensitivity	Sensitivity is lowest for the stage 0-I disease screening must catch	Combine orthogonal analytes to raise early-stage sensitivity at fixed high specificity	Adequately powered case-control, then prospective evaluation	Specificity fixed for a low-prevalence setting; adequate events per variable
Integration (multiomics)	Separate analyte streams; overfitting in small cohorts	Rigorously develop and externally validate integrated panels before claiming readiness	Multi-cohort development with independent external validation	Sufficient sample size; prespecified plans; transparent reporting (TRIPOD+AI)
Risk-stratified triage	Models judged by discrimination alone; pathway position and intended use often unclear	Embed biomarkers in calibrated triage pathways and evaluate net benefit	Prospective implementation with decision-curve/net-benefit analysis	Calibration and net benefit, not the area under the curve alone
Clinical utility and health economics	Utility unproven; downstream diagnostic cascade may be costly or harmful	Test surviving candidates in pragmatic trials with health-economic evaluation	Pragmatic randomized or well-designed comparative implementation study	Clinically meaningful endpoints (stage shift, mortality, avoided endoscopy); cost-effectiveness
Standardization and reporting	Pre-analytical variability; high risk of bias; poor reporting	Standardize pre-analytics and adopt reporting and bias standards	Harmonized multi-site protocols and shared reference materials	STARD, TRIPOD+AI and REMARK reporting; QUADAS-2/PROBAST risk-of-bias appraisal; matched leukocyte sequencing for mutation assays

## Abbreviations

ESCC: esophageal squamous cell carcinoma
EAC: esophageal adenocarcinoma
BE: Barrett esophagus
HGD: high-grade dysplasia
cfDNA: cell-free DNA
ctDNA: circulating tumor DNA (the tumor-derived fraction of cfDNA)
EV: extracellular vesicle
miRNA: microRNA
MCED: multicancer early detection
TFF3: trefoil factor 3
AUROC: area under the receiver operating characteristic curve
PPV: positive predictive value
NPV: negative predictive value
DCA: decision-curve analysis
GERD: gastroesophageal reflux disease
GLOBOCAN: Global Cancer Observatory database
