# Supplementary material for: Liquid Biopsy and Nonendoscopic Biomarkers for Precision Early Detection of Esophageal Cancer: Evidence, Risk Stratification and Translational Challenges
Source: J Cancer. 2026 Sep 3;17(9):1639–52. doi: 10.7150/jca.140909 (PMC13573304; doi:10.7150/jca.140909)
Supplement: Supplementary file 1 [file jcav17p1639s1.pdf]

Supplementary Material

Table S1. Detailed evidence matrix of studies of early detection, nonendoscopic sampling, liquid biopsy and risk prediction in esophageal cancer. Studies are classified by subtype and intended use; reference numbers correspond to the main manuscript reference list.

Study (ref.)	Country	Population / subtype / intended use	Sample type	Biomarker, tool or model	Study design	Validation status	Key findings (source-reported)	Risk-of-bias / applicability limitation
Wong MCS 2022 [3]	-	ESCC<br>Intended use: Diagnostic evaluation in clinically recruited participants; screening utility unproven	N/A (multiple screening modalities including serum biomarkers)	Autoantibody panel	Systematic review / meta-analysis	Evidence synthesis	We included 161 studies conducted in 81 research articles involving 32,209 subjects.	Between-study heterogeneity and variable study quality
Chen R 2020 [9]	China	pan-GI<br>Intended use: Population screening / outcome evaluation in high-incidence settings	N/A (endoscopy)	-	Cohort study	Cohort	Our cohort analysis included 637 500 people: 299 483 in the control group and 338 017 in the invited to screening group, 113 340 (33.53%) of whom were screened eventually.	External/prospective validation required
He Z 2018 [10]	China	ESCC<br>Intended use: Population screening / outcome evaluation in high-incidence settings	N/A (Lugol chromoendoscopy)	-	Randomized or pragmatic trial	Randomized / pragmatic evaluation	A total of 17 151 and 16 797 participants were enrolled in screening and control arms from January 2012 to September 2016.	Outcome applies only to the stated intervention and population; long-term cancer utility may remain uncertain
Xia C 2024 [11]	Multicenter	pan-GI<br>Intended use: Population screening / outcome evaluation in high-incidence settings	N/A (endoscopy)	-	Randomized or pragmatic trial	Randomized / pragmatic evaluation	A total of 234,635 participants were included in the intention-to-screen analysis, with a median age of 52 years.	Outcome applies only to the stated intervention and population; long-term cancer utility may remain uncertain
Wei WQ 2020 [12]	China	ESCC<br>Intended use: Population screening / outcome evaluation in high-incidence settings	tissue (background; endoscopic biopsy histology)	-	Prospective cohort or diagnostic study	Prospective evaluation; external utility not established	We identified 143 new ESCC cases (0.68%) and 62 ESCC deaths (0.29%) during a median follow-up of 8.5 years.	Applicability beyond the study setting and effect on clinical outcomes require confirmation
Li H 2023 [13]	China	ESCC<br>Intended use: Population screening / outcome evaluation in high-incidence settings	N/A (endoscopy)	-	Cohort study	Cohort	A total of 42,827 participants (40,977 with negative endoscopy findings, 1562 with mild dysplasia, and 288 with moderate dysplasia) were included.	External/prospective validation required

Study (ref.)	Country	Population / subtype / intended use	Sample type	Biomarker, tool or model	Study design	Validation status	Key findings (source-reported)	Risk-of-bias / applicability limitation
Hvid-Jensen F 2011 [14]	-	Barrett/precursor Intended use: Targeted case finding for previously undiagnosed Barrett esophagus	N/A	-	Cohort study	Cohort	11,028 patients with Barrett's esophagus, median 5.2 years follow-up. Incidence rate for adenocarcinoma 1.2 cases per 1000 person-years (95% CI, 0.9 to 1.5).	External/prospective validation required
Qiao Y 2015 [15]	-	Barrett/precursor Intended use: Targeted case finding for previously undiagnosed Barrett esophagus	N/A	-	Systematic review / meta-analysis	Evidence synthesis	51 studies with 11,028 subjects. Pooled EAC incidence 5.5 per 1,000 person-years (95% CI 4.2-6.8); pooled EAC/HGD incidence 7.7 (95% CI 5.7-9.7).	Between-study heterogeneity and variable study quality
Redston M 2022 [16]	-	Barrett/precursor Intended use: Established-Barrett surveillance or progression prediction	tissue(background)	-	Observational or biomarker study	Prospective evaluation; external utility not established	Criteria developed and validated by sequencing; tested retrospectively in 561 patients and prospectively in 1487 patients.	Generalizability beyond setting
Shaheen NJ 2022 [17]	-	Barrett/precursor Intended use: Targeted case finding for previously undiagnosed Barrett esophagus	N/A	-	Clinical practice guideline	Not applicable (guideline)	Clinical practice guideline (GRADE).	Not primary performance evidence; recommendations are population- and evidence-specific
Wani S 2025 [18]	-	Barrett/precursor Intended use: Targeted case finding for previously undiagnosed Barrett esophagus	N/A	-	Clinical practice guideline	Not applicable (guideline)	Clinical practice guideline (GRADE).	Not primary performance evidence; recommendations are population- and evidence-specific
Weusten BLAM 2023 [19]	-	Barrett/precursor Intended use: Targeted case finding for previously undiagnosed Barrett esophagus	N/A	-	Clinical practice guideline	Not applicable (guideline)	Clinical practice guideline.	Not primary performance evidence; recommendations are population- and evidence-specific
Nguyen TH 2020 [20]	-	Barrett/precursor Intended use: Targeted case finding for previously undiagnosed Barrett esophagus	tissue(background)	-	Prospective cohort or diagnostic study	Prospective evaluation; external utility not established	44 BE cases and 469 controls (prevalence 8.6%). Among 371 patients without GERD symptoms, 25 (6.7%) had BE. AGA guidelines (>=2 BE risk factors): sensitivity 100%, specificity 0.2%.	Applicability beyond the study setting and effect on clinical outcomes require confirmation

Study (ref.)	Country	Population / subtype / intended use	Sample type	Biomarker, tool or model	Study design	Validation status	Key findings (source-reported)	Risk-of-bias / applicability limitation
Ross-Innes CS 2015 [21]	Multicenter	Barrett/precursor Intended use: Targeted case finding for previously undiagnosed Barrett esophagus	Cytosponge	-	Case-control diagnostic study	Development or internal validation	1,110 individuals (463 controls with dyspepsia/reflux symptoms and 647 BE cases); 1,042 (93.9%) successfully swallowed the Cytosponge.	Spectrum bias and enriched prevalence; intended-use screening performance uncertain
Fitzgerald RC 2020 [22]	Multicenter	Barrett/precursor Intended use: Targeted case finding for previously undiagnosed Barrett esophagus	Cytosponge	-	Randomized or pragmatic trial	Randomized / pragmatic evaluation	13,657 eligible patients identified; 13,514 randomised (usual care n=6531; intervention n=6983).	External/prospective validation required
Kakar MT 2025 [23]	-	Barrett/precursor Intended use: Targeted case finding for previously undiagnosed Barrett esophagus	Cytosponge	-	Systematic review / meta-analysis	Evidence synthesis	Six studies comprising 3438 participants.	Between-study heterogeneity and variable study quality
Gao Y 2023 [24]	-	both Intended use: Background, guideline or methodological evidence	sponge cytology (Cytosponge-type)	-	Prospective cohort or diagnostic study	Prospective evaluation; external utility not established	17,498 eligible participants involved in model training and validation.	Applicability beyond the study setting and effect on clinical outcomes require confirmation
Moinova HR 2018 [25]	-	Barrett/precursor Intended use: Targeted case finding for previously undiagnosed Barrett esophagus	encapsulated balloon (EsoCheck) esophageal cell sample	-	Observational or biomarker study	Internal validation	DNA methylation tested in cytology brushings of the distal esophagus from 173 individuals with or without BE.	External/prospective validation required
Shaheen NJ 2025 [26]	Multicenter	Barrett/precursor Intended use: Targeted case finding for previously undiagnosed Barrett esophagus	EsoCheck balloon (EsoGuard methylated DNA assay)	-	Prospective cohort or diagnostic study	Prospective evaluation; external utility not established	163/180 (90.6%) had EGD and successful EC administration; of 122 samples analyzed, 93 contributed to primary endpoint; 8 subjects (8.6%) had BE on EGD.	Applicability beyond the study setting and effect on clinical outcomes require confirmation
Pilonis ND 2022 [27]	Multicenter	Barrett/precursor Intended use: Targeted case finding for previously undiagnosed Barrett esophagus	Cytosponge	-	Observational or biomarker study	Prospective evaluation; external utility not established	Prevalence of high-grade dysplasia or cancer 17% (92/557) training, 10% (35/344) validation.	External/prospective validation required

Study (ref.)	Country	Population / subtype / intended use	Sample type	Biomarker, tool or model	Study design	Validation status	Key findings (source-reported)	Risk-of-bias / applicability limitation
Gehrung M 2021 [28]	-	Barrett/precursor Intended use: Targeted case finding for previously undiagnosed Barrett esophagus	Cytosponge (TFF3/H&E slides)	-	Observational or biomarker study	Randomized / pragmatic evaluation	Trained and independently validated on data from two clinical trials, analyzing a combined total of 4,662 pathology slides from 2,331 patients. Approach defines eight triage classes.	External/prospective validation required
Liu MC 2020 [29]	-	pan-cancer Intended use: MCED contextual evidence (indirect, not esophageal-specific validation)	plasma cfDNA	Multi-cancer assay	Prospective cohort or diagnostic study	Prospective evaluation; external utility not established	The 6689 participants [2482 cancer (>50 cancer types), 4207 non-cancer] were divided into training and validation sets. In validation, specificity was 99.3% [95% CI: 98.3% to 99.8%.	Indirect evidence; esophageal-specific subtype performance and PPV not established
Klein EA 2021 [30]	-	pan-cancer Intended use: MCED contextual evidence (indirect, not esophageal-specific validation)	plasma cfDNA	Multi-cancer assay	Prospective cohort or diagnostic study	Prospective evaluation; external utility not established	This pre-specified substudy included 4077 participants in an independent validation set (cancer: n = 2823; non-cancer: n = 1254).	Indirect evidence; esophageal-specific subtype performance and PPV not established
Razavi P 2019 [31]	-	general/methods Intended use: Diagnostic evaluation in clinically recruited participants; screening utility unproven	plasma cfDNA + matched white blood cell DNA	ctDNA/fragmento mics	Prospective cohort or diagnostic study	Prospective evaluation; external utility not established	High-intensity sequencing assay of cfDNA and matched white blood cell DNA covering 508 genes; 2 megabases.	Applicability beyond the study setting and effect on clinical outcomes require confirmation
Liu J 2024 [32]	-	ESCC Intended use: Diagnostic evaluation in clinically recruited participants; screening utility unproven	plasma cfDNA	cfDNA methylation	Case-control diagnostic study	Development or internal validation	Whole-genome bisulfite sequencing on 460 cfDNA samples. cfDNA methylation markers detectable in 70% of ESCCs and 50% of precancerous lesions.	Spectrum bias and enriched prevalence; intended-use screening performance uncertain
Zhang R 2025 [33]	Multicen ter	both Intended use: Diagnostic evaluation in clinically recruited participants; screening utility unproven	plasma cfDNA	cfDNA methylation	Prospective cohort or diagnostic study	Randomized / pragmatic evaluation	3-MDM diagnostic model discriminated EC from non-EC in a multicenter prospective cohort (n=1,429) with sensitivity 85.5% and specificity 95.3%.	Applicability beyond the study setting and effect on clinical outcomes require confirmation

Study (ref.)	Country	Population / subtype / intended use	Sample type	Biomarker, tool or model	Study design	Validation status	Key findings (source-reported)	Risk-of-bias / applicability limitation
Bian Y 2024 [34]	HUNT	both<br>Intended use: Diagnostic evaluation in clinically recruited participants; screening utility unproven	plasma cfDNA	cfDNA methylation	Prospective cohort or diagnostic study	Prospective evaluation; external utility not established	Prospectively enrolled 1116 participants (334 EC, 71 HGIN, 711 controls). AUC for detecting EC 0.903 (95% CI 0.880-0.927) and HGIN 0.727 (95% CI 0.653-0.801).	Applicability beyond the study setting and effect on clinical outcomes require confirmation
Huang A 2025 [35]	Multicenter	pan-GI<br>Intended use: Diagnostic evaluation in clinically recruited participants; screening utility unproven	plasma cfDNA	ctDNA/fragmentomics	Prospective cohort or diagnostic study	Prospective evaluation; external utility not established	GutSeer was trained and validated using plasma from 1,057 cancer patients and 1,415 non-cancer controls; the locked model was blindly tested in an independent cohort of 846 participants.	Applicability beyond the study setting and effect on clinical outcomes require confirmation
Ma C 2025 [36]	-	both<br>Intended use: Diagnostic evaluation in clinically recruited participants; screening utility unproven	plasma/serum cfDNA (pooled)	cfDNA methylation	Systematic review / meta-analysis	Evidence synthesis	A total of 14 studies with a total of 3,936 patients were included.	Between-study heterogeneity and variable study quality
Yu M 2022 [37]	-	EAC<br>Intended use: Background, guideline or methodological evidence	tissue biopsy and endoscopic brushing (framed as detection biomarkers)	cfDNA methylation	Observational or biomarker study	Internal validation	Five candidate methylation markers were significantly hypermethylated in HGD/EAC samples compared with SQ or NDBE ($P < 0.01$).	Not primary performance evidence; recommendations are population- and evidence-specific
Cristiano S 2019 [38]	-	pan-cancer<br>Intended use: MCED contextual evidence (indirect, not esophageal-specific validation)	plasma cfDNA (WGS)	ctDNA/fragmentomics	Case-control diagnostic study	Development or internal validation	Analysed fragmentation profiles of 236 patients with breast, colorectal, lung, ovarian, pancreatic, gastric or bile duct cancer and 245 healthy individuals.	Indirect evidence; esophageal-specific subtype performance and PPV not established
Jiao Z 2024 [39]	-	ESCC<br>Intended use: Diagnostic evaluation in clinically recruited participants; screening utility unproven	plasma cfDNA	ctDNA/fragmentomics	Observational or biomarker study	Independent or external validation reported	Diagnostic model development / validation (stacked ensemble machine-learning model on cfDNA WGS fragmentomics; independent and external validation cohorts).	External/prospective validation required

Study (ref.)	Country	Population / subtype / intended use	Sample type	Biomarker, tool or model	Study design	Validation status	Key findings (source-reported)	Risk-of-bias / applicability limitation
Chen X 2020 [40]	-	pan-cancer Intended use: MCED contextual evidence (indirect, not esophageal-specific validation)	plasma cfDNA	cfDNA methylation	Nested case-control study	Prospective evaluation; external utility not established	PanSeer detects five common types of cancer in 88% (95% CI: 80-93%) of post-diagnosis patients with a specificity of 96% (95% CI: 93-98%).	Indirect evidence; esophageal-specific subtype performance and PPV not established
Zhang C 2010 [41]	-	ESCC Intended use: Diagnostic evaluation in clinically recruited participants; screening utility unproven	serum	Circulating miRNA/ncRNA	Observational or biomarker study	Internal validation	Serum samples from 290 ESCC patients and 140 age- and sex-matched controls; Solexa sequencing screened 141 patients and 40 controls.	Spectrum bias; needs prospective validation
Zhang WT 2023 [42]	-	both Intended use: Diagnostic evaluation in clinically recruited participants; screening utility unproven	serum/plasma (pooled)	Circulating miRNA/ncRNA	Systematic review / meta-analysis	Evidence synthesis	85 studies from 50 articles included.	Between-study heterogeneity and variable study quality
Tong YS 2015 [43]	-	ESCC Intended use: Diagnostic evaluation in clinically recruited participants; screening utility unproven	plasma	Circulating miRNA/ncRNA	Observational or biomarker study	Development or internal validation	Ten candidate lncRNAs screened. Plasma levels of POU3F3, HNF1A-AS1 and SPRY4-IT1 significantly higher in ESCC than controls. Plasma POU3F3 provided highest diagnostic performance (AUC 0.842.	Spectrum bias; needs prospective validation
Petrick JL 2021 [44]	-	EAC Intended use: Prediagnostic risk or early-detection research	serum	Circulating miRNA/ncRNA	Nested case-control study	Prediagnostic / nested evaluation	All 62 EA cases from the US Multi-Center case-control study, serum matched 1:1 to controls. MiRNAs (n = 2064) assessed via HTG EdgeSeq.	Spectrum bias and enriched prevalence; intended-use screening performance uncertain
Li K 2023 [45]	-	ESCC Intended use: Diagnostic evaluation in clinically recruited participants; screening utility unproven	saliva (extracellular vesicles/particl es)	Circulating miRNA/ncRNA	Cohort study	Independent or external validation reported	Salivary EVP miRNA profiled in a pilot cohort (n = 54); candidates measured in a discovery cohort (n = 72).	External/prospective validation required

Study (ref.)	Country	Population / subtype / intended use	Sample type	Biomarker, tool or model	Study design	Validation status	Key findings (source-reported)	Risk-of-bias / applicability limitation
Chen J 2025 [46]	Multicenter	ESCC<br>Intended use: Diagnostic evaluation in clinically recruited participants; screening utility unproven	serum glycosylated (fucosylated/sialylated) EVs	-	Case-control diagnostic study	Independent or external validation reported	A total of 371 ESCC and 303 healthy controls (HCs) were recruited in this multi-stage, multicentre case-control study.	Spectrum bias and enriched prevalence; intended-use screening performance uncertain
Chiam K 2020 [47]	-	EAC<br>Intended use: Diagnostic evaluation in clinically recruited participants; screening utility unproven	serum and plasma small EVs	-	Observational or biomarker study	Internal validation	Matched serum and plasma samples were obtained from 10 healthy controls and 10 patients with esophageal adenocarcinoma.	External/prospective validation required
Xu YW 2014 [48]	-	ESCC<br>Intended use: Diagnostic evaluation in clinically recruited participants; screening utility unproven	serum autoantibody (ELISA)	Autoantibody panel	Cohort study	Internal validation	We assessed serum autoantibodies in 513 participants: 388 with ESCC and 125 normal controls. The validation cohort comprised 371 participants: 237 with ESCC, and 134 normal controls.	Spectrum bias; needs prospective validation
Zhang H 2015 [49]	-	both<br>Intended use: Diagnostic evaluation in clinically recruited participants; screening utility unproven	serum autoantibody (background/synthesis)	Autoantibody panel	Systematic review / meta-analysis	Evidence synthesis	Overall, 45 articles reporting the detection of 35 different autoantibodies met the inclusion criteria of this review.	Between-study heterogeneity and variable study quality
Xu YW 2019 [50]	-	both<br>Intended use: Diagnostic evaluation in clinically recruited participants; screening utility unproven	serum autoantibody (background)	Autoantibody panel	Narrative review	Descriptive	Narrative review.	External/prospective validation required
Zhao Y 2024 [51]	Multicenter	ESCC<br>Intended use: Diagnostic evaluation in clinically recruited participants; screening utility unproven	serum/urine (matched tissue as background)	-	Observational or biomarker study	Descriptive	Here we conduct NMR- and MS-based metabolomics on 1,153 matched ESCC tissues, normal mucosae, pre- and one-week post-operative sera and urines from 560 participants across three hospitals, with machine learning and WGCNA.	External/prospective validation required

Study (ref.)	Country	Population / subtype / intended use	Sample type	Biomarker, tool or model	Study design	Validation status	Key findings (source-reported)	Risk-of-bias / applicability limitation
Yuan Y 2021 [52]	-	ESCC<br>Intended use: Diagnostic evaluation in clinically recruited participants; screening utility unproven	serum (lipidomics)	-	Observational or biomarker study	Internal validation	A total of 525 serum samples were subjected to lipidomic analysis.	External/prospective validation required
Liu M 2024 [53]	-	ESCC<br>Intended use: Prediagnostic risk or early-detection research	serum (metabolomics)	-	Nested case-control study	Prediagnostic / nested evaluation	Nested case-control study based on two community-based screening cohorts.	Spectrum bias and enriched prevalence; intended-use screening performance uncertain
Li S 2024 [54]	UK Biobank	pan-GI<br>Intended use: Diagnostic evaluation in clinically recruited participants; screening utility unproven	plasma (metabolomics)	-	Prospective cohort or diagnostic study	Prospective evaluation; external utility not established	A total of 15 metabolites were identified as UGI-cancer-related differential metabolites.	Applicability beyond the study setting and effect on clinical outcomes require confirmation
Antonowicz S 2015 [55]	-	pan-GI<br>Intended use: Diagnostic evaluation in clinically recruited participants; screening utility unproven	blood (serum/plasma metabolomics)	-	Systematic review / meta-analysis	Evidence synthesis	Twenty-nine studies (15 colorectal, 9 esophageal, 3 gastric, and 2 mixed) with data from 10,835 participants were included. All reported significant differences in hematologic metabolites.	Between-study heterogeneity and variable study quality
Xie SH 2020 [56]	-	EAC<br>Intended use: Diagnostic evaluation in clinically recruited participants; screening utility unproven	serum/plasma (circulating inflammatory/ metabolic biomarkers)	-	Systematic review / meta-analysis	Evidence synthesis	Among 7,641 studies, 19 were eligible for inclusion (12 cross-sectional, two nested case-control, and five cohort studies).	Outcome applies only to the stated intervention and population; long-term cancer utility may remain uncertain
Peters BA 2017 [57]	-	both<br>Intended use: Prediagnostic risk or early-detection research	saliva	Microbiome	Nested case-control study	Prospective evaluation; external utility not established	Oral bacteria were assessed using 16S rRNA gene sequencing in prediagnostic mouthwash samples from n = 81/160 EAC and n = 25/50 ESCC cases/matched controls.	Spectrum bias and enriched prevalence; intended-use screening performance uncertain
Liu F 2020 [58]	China	ESCC<br>Intended use: Prediagnostic risk or early-detection research	saliva	Microbiome	Nested case-control study	Prediagnostic / nested evaluation	428 pre-diagnostic oral specimens from 84 cases with esophageal lesions of severe squamous dysplasia and above (SDA) and 168 matched healthy controls.	Spectrum bias and enriched prevalence; intended-use screening performance uncertain

Study (ref.)	Country	Population / subtype / intended use	Sample type	Biomarker, tool or model	Study design	Validation status	Key findings (source-reported)	Risk-of-bias / applicability limitation
Snider EJ 2019 [59]	-	EAC<br>Intended use: Background, guideline or methodological evidence	tissue(backgrou nd)	Microbiome	Case-control diagnostic study	Development or internal validation	A total of 45 patients were enrolled and included in the analyses [16 controls; 14 Barrett's esophagus without dysplasia (NDBE); 6 low-grade dysplasia (LGD); 5 high-grade dysplasia (HGD).	Spectrum bias and enriched prevalence; intended-use screening performance uncertain
Chen W 2020 [60]	China	ESCC<br>Intended use: Pre-endoscopy population risk triage	N/A (questionnaire-based risk model)	-	Observational or biomarker study	Cohort	In total, 86 745 participants enrolled in a population-based esophageal cancer screening program in rural China between 2007 and 2012 were included and followed up until December 31, 2015.	Calibration, pathway position and transportability must be evaluated
Han J 2023 [61]	Multicen ter	ESCC<br>Intended use: Background, guideline or methodological evidence	N/A	-	Prospective cohort or diagnostic study	Prospective evaluation; external utility not established	In this diagnostic study of 104 129 participants (56.39% women; mean [SD] age, 54.31 [7.64] years), 59 481 formed the derivation set and 44 648 formed the validation set.	Applicability beyond the study setting and effect on clinical outcomes require confirmation
Jiang H 2023 [62]	Multicen ter	ESCC<br>Intended use: Background, guideline or methodological evidence	N/A	-	Systematic review / meta-analysis	Evidence synthesis	External validation in a multicenter prospective cohort of 89,753 participants aged 40-69 years (first endoscopy April 2017-March 2021, followed to December 31, 2022).	Between-study heterogeneity and variable study quality
Wang QL 2021 [63]	HUNT	ESCC<br>Intended use: Background, guideline or methodological evidence	N/A	-	Model or assay study with external validation	Independent or external validation reported	Model derived on the Nord-Trondelag Health Study (HUNT) and validated on the UK Biobank cohort. The developed risk prediction model included age, sex, smoking, alcohol, and BMI.	Not primary performance evidence; recommendations are population- and evidence-specific
Xie SH 2018 [64]	Norway	EAC<br>Intended use: Background, guideline or methodological evidence	N/A	-	Prospective cohort or diagnostic study	Prospective evaluation; external utility not established	Population-based cohort of 62,576 participants (HUNT, Norway); during 1,085,137 person-years of follow-up, 29 incident cases of OAC occurred.	Applicability beyond the study setting and effect on clinical outcomes require confirmation
Nguyen TH 2022 [65]	-	Barrett/precursor<br>Intended use: Targeted case finding for previously undiagnosed Barrett esophagus	N/A	-	Model or assay study with external validation	Independent or external validation reported	Risk of EAC in those with BE is 11-fold greater than the general population. Among 608 BE patients, 24 progressed to HGD/EAC.	External/prospective validation required

Study (ref.)	Country	Population / subtype / intended use	Sample type	Biomarker, tool or model	Study design	Validation status	Key findings (source-reported)	Risk-of-bias / applicability limitation
Schröder J 2022 [66]	-	EAC<br>Intended use: Background, guideline or methodological evidence	N/A	-	Systematic review / meta-analysis	Evidence synthesis	Combined data increasing the sample size to 16,790 BE/EA cases and 32,476 controls. The GWAS meta-analysis identified 27 BE and/or EA risk loci, 11 of which were novel.	Between-study heterogeneity and variable study quality
Iyer PG 2023 [67]	-	EAC<br>Intended use: Background, guideline or methodological evidence	N/A	-	Case-control diagnostic study	Development or internal validation	Using the Clinical Data Analytics Platform (deidentified EHR of 6 million Mayo Clinic patients), identified 8,476 BE cases, 1,539 EAC cases, and 252,276 controls.	Spectrum bias and enriched prevalence; intended-use screening performance uncertain
Li H 2021 [68]	-	both<br>Intended use: Background, guideline or methodological evidence	N/A	-	Systematic review / meta-analysis	Evidence synthesis	Of the 13 models included in the qualitative analysis, 8 were developed for esophageal squamous cell carcinoma (ESCC) and the other 5 were developed for esophageal adenocarcinoma (EAC).	Between-study heterogeneity and variable study quality
Wolff RF 2019 [69]	-	general/methods<br>Intended use: Background, guideline or methodological evidence	N/A	-	Observational or biomarker study	Descriptive	PROBAST was informed by a Delphi procedure involving 38 experts.	Not primary performance evidence; recommendations are population- and evidence-specific
Vickers AJ 2016 [70]	-	general/methods<br>Intended use: Background, guideline or methodological evidence	N/A	-	Observational or biomarker study	Descriptive	Methodological/educational article on decision-analytic net benefit.	Not primary performance evidence; recommendations are population- and evidence-specific
Xia R 2021 [76]	China	ESCC<br>Intended use: Background, guideline or methodological evidence	N/A	-	Observational or biomarker study	Descriptive	Compared with no screening, as the score threshold lowered, gained QALYs increased, with 49 to 172 QALYs and 329 to 1147 QALYs gained from screening at 40 and 65 years, respectively.	Not primary performance evidence; recommendations are population- and evidence-specific
Swart N 2021 [77]	-	Barrett/precursor<br>Intended use: Targeted case finding for previously undiagnosed Barrett esophagus	Cytosponge	-	Observational or biomarker study	Descriptive	Per person, one round of Cytosponge-TFF3 screening (with confirmatory endoscopy and treatment) costed £82 more than usual care and generated an additional 0.015 QALYs, at an ICER of £5,500 per QALY gained.	Model assumptions

Study (ref.)	Country	Population / subtype / intended use	Sample type	Biomarker, tool or model	Study design	Validation status	Key findings (source-reported)	Risk-of-bias / applicability limitation
Benaglia T 2012 [78]	-	Barrett/precursor Intended use: Targeted case finding for previously undiagnosed Barrett esophagus	Cytosponge	-	Observational or biomarker study	Cohort	Hypothetical cohort of 50-year-old UK men with GERD, assumed Barrett's esophagus prevalence 8%; invitation acceptance rates 23% (endoscopy) and 45% (Cytosponge).	Model assumptions
Schrag D 2023 [79]	-	pan-cancer Intended use: MCED contextual evidence (indirect, not esophageal-specific validation)	plasma cfDNA	Multi-cancer assay	Prospective cohort or diagnostic study	Prospective evaluation; external utility not established	Between Dec 12, 2019, and Dec 4, 2020, we recruited 6662 participants.	Indirect evidence; esophageal-specific subtype performance and PPV not established
Nicholson BD 2023 [80]	Multicenter	pan-cancer (symptomatic; gynaecological/lung/ upper and lower GI referrals) Intended use: MCED contextual evidence (indirect, not esophageal-specific validation)	plasma cfDNA	Multi-cancer assay	Prospective cohort or diagnostic study	Prospective evaluation; external utility not established	Of 5851 clinically evaluable participants, 5461 were included in the final cohort (368 [6.7%] with a cancer diagnosis and 5093 [93.3%] without).	Indirect evidence; esophageal-specific subtype performance and PPV not established
